# Transcriptome sequencing and metabolome analysis reveal the metabolic reprogramming of partial hepatectomy and extended hepatectomy

**DOI:** 10.1186/s12864-023-09647-0

**Published:** 2023-09-07

**Authors:** Zeyuan Li, Bo Peng, Shilian Chen, Jiaping Li, Kai Hu, Lijuan Liao, Qiuli Xie, Mei Yao, Lixing Liang, Stephen Tomlinson, Guandou Yuan, Songqing He

**Affiliations:** 1 Division of Hepatobiliary Surgery, the First Affiliated Hospital of Guangxi Medical University, NO 6 Shuangyong Road, Nanning, Guangxi 530021 China; 2 Key Laboratory of Early Prevention and Treatment for Regional High Frequency Tumor, Guangxi Medical University, Ministry of Education, Nanning, Guangxi 530021 China; 3 Guangxi Key Laboratory of Immunology and Metabolism for Liver Diseases, the First Affiliated Hospital of Guangxi Medical University, Nanning, Guangxi 530021 China; 4 Guangxi Key Laboratory of Precision Medicine in Cardio-cerebrovascular Diseases Control and Prevention, the First Affiliated Hospital of Guangxi Medical University, Nanning, Guangxi 530021 China; 5 Department of Radiation Oncology, the First Affiliated Hospital of Guangxi Medical University, Nanning, Guangxi 530021 China; 6 Department of Microbiology and Immunology, Medical University of South Carolina, Charleston, SC USA

**Keywords:** Partial hepatectomy, Extended hepatectomy, Transcriptome, Metabolome, Regeneration

## Abstract

Surgical resection remains a critical treatment option for many patients with primary and secondary hepatic neoplasms. Extended hepatectomy (eHx) may be required for some patients with large tumors, which may cause liver failure and death. Partial hepatectomy (pHx) and eHx mouse models were constructed, liver tissues were sampled at 18, 36, and 72 h posthepatectomy. Transcriptome and metabolome analyses were employed to explore the different potential mechanisms in regeneration and injury between pHx and eHx. The results showed that eHx was associated with more severe liver injury and lower survival rates than pHx. Transcriptomics data showed there were 1842, 2129, and 1277 differentially expressed genes (DEGs) in eHx and 962, 1305, and 732 DEGs in pHx at 18, 36, and 72 h posthepatectomy, respectively, compared with the those in the sham groups. Compared with pHx, the number of DEGs in the eHx group reached a maximum of 230 at 18 h after surgery and decreased sequentially to 87 and 43 at 36 and 72 h. Metabolomics analysis identified a total of 1399 metabolites, and 48 significant differentially produced metabolites (DPMs) were screened between eHx and pHx. Combined analysis of DEGs and DPMs indicated that cholesterol metabolism and insulin resistance may be two important pathways for liver regeneration and mouse survival postextended hepatectomy. Our results showed the global influence of pHx and eHx on the transcriptome and metabolome in mouse liver, and revealed cholesterol metabolism and insulin resistance pathways might be involved in regeneration post-pHx and -eHx.

**Supplementary Information:**

The online version contains supplementary material available at 10.1186/s12864-023-09647-0.

## Introduction

The liver has the extraordinary ability to regenerate to its original size after injury or surgical resection. Successful regeneration is essential for the morphological and functional recovery of the remnant liver. The stability of liver function and size is essential for whole body homeostasis [1, 2]. The process by which the liver adjusts its size to 100% has been called “hepatostat” [3]. The cellular and molecular mechanisms involved in liver injury and regeneration posthepatectomy have been intensely studied for decades. It is known that the process of liver regeneration posthepatectomy is broadly defined by three distinct phases: initiation phase, proliferation phase, and termination phase [4]. Transcription factors are activated to promote hepatocyte division in the priming phase, and cell cycle entry approximately 16–20 h post pHx. DNA replication and hepatic division are the main action in proliferation phase, and the peak of DNA replication and cell size occurs at 36 h after pHx. Cell size peaks at 36 h after pHx. Finally, organ size is controlled by the regulation of cell division and apoptosis in the termination phase, and most of the increase in liver mass occurs by 72 h after pHx [4–7]. Liver regeneration requires the activity of multiple signaling pathways [7], but the roles and functions of these various processes are not fully understood.

Clinically, surgical resection remains a critical treatment option for many patients with primary and secondary hepatic neoplasms [8]. Extended hepatectomy (eHx) may be required for some patients with large tumors, which could cause fatal postoperative complications, including liver failure and death [9, 10]. In fact, regeneration failure occurs frequently in patients with acute liver failure or after extensive liver resection [11]. The mechanisms underlying liver regeneration failure posthepatectomy in some patients remain poorly understood. Further research aimed at understanding the mechanisms of liver regeneration could identify new therapeutic targets to improve patient outcomes and reduce the possibility of liver failure after hepatectomy.

Currently, it is deemed safe to resect approximately 70% of a healthy liver. Liver resections beyond 70% markedly increase the risk of liver failure, and the fatality rate is unacceptably high [8]. To ensure the safety and prognosis of patients, patients with normal liver function maintain > 25–30% of their pre-operative liver volume, while patients with livers that are cirrhotic, cholestatic, steatotic, or injured by chemotherapy maintain > 40% of their preoperative liver volume [12]. EHx may cause liver failure and individual death. A significant reduction in liver mass increases the patient’s risk of small-for-size syndrome (SFSS), which is a syndrome caused when the remaining liver or liver graft cannot meet the metabolic demands of the recipient [13, 14]. Liver failure often develops from SFSS. The molecular processes underlying liver failure are still unclear [15]. Partial hepatectomy (pHx, approximately 70%) does not cause the death of mice, but eHx (approximately 86%) may cause liver failure [16–18]. Thus, this model is suitable for exploring the potential mechanisms of liver regeneration and failure associated with eHx and pHx. A mouse model has been widely used for the study of liver regeneration after pHx, which has been proven to be accessible and practical in these experimental settings [19, 20]. In addition, extended hepatectomy was designed to investigate primary liver regeneration in a small liver remnant to better understand SFSS [10].

Multiomics analysis technologies are frequently used to explore the pathogenesis and mechanisms associated with different kinds of diseases. The comprehensive analysis of different types of omics data could provide perspective into the pathogenesis of diseases [21, 22]. In addition, multiomics analyses are often used to elucidate potential global changes in research objects and screen candidate molecules for further studies [23]. Multiomics analyses include transcriptomic analysis and metabolomics analysis. Transcriptomic analysis of mouse liver tissue at 1, 8, 16, 32, and 48 h post pHx and eHx showed that the regenerative process requires transient activation and silencing of approximately two dozen of intracellular signalling pathways [24]. Transcriptomic analysis of mouse liver tissue at 0 and 36 h post pHx indicated that liver regeneration was involved in cell cycle regulation, material metabolism, and multiple classical pathways related to the DNA synthesis process [25]. A combination of integrated transcriptomic and metabolomic analyses suggests cell division leads to hepatic metabolic remodelling during liver regeneration post pHx [4]. Currently, the metabolome analysis of eHx in mice was lacking. Integrated analysis of transcriptome and metabolome data of pHx and eHx may provide insight into the characteristics and mechanisms underlying liver injury and regeneration posthepatectomy. Therefore, it is feasible to use multiomics analysis to study the mechanism of liver regeneration dysfunction caused by extended hepatectomy and to provide a theoretical basis for improving the prognosis of patients with large-volume hepatectomy in clinical practice.

In this study, to characterize the three distinct phases of regeneration posthepatectomy, mouse livers were sampled at 18 h, 36 and 72 h after pHx and eHx at different time points posthepatectomy. A comprehensive analysis of transcriptomics and metabolomics was performed to study the transcriptional reprogramming and metabolic changes of liver injury and repair. To further investigate the difference in the metabolic alterations and molecular mechanisms of pHx and eHx, systemic data analysis was performed with both omics datasets.

## Results

### Survival and pathological features after hepatectomy

**Fig. 1:**
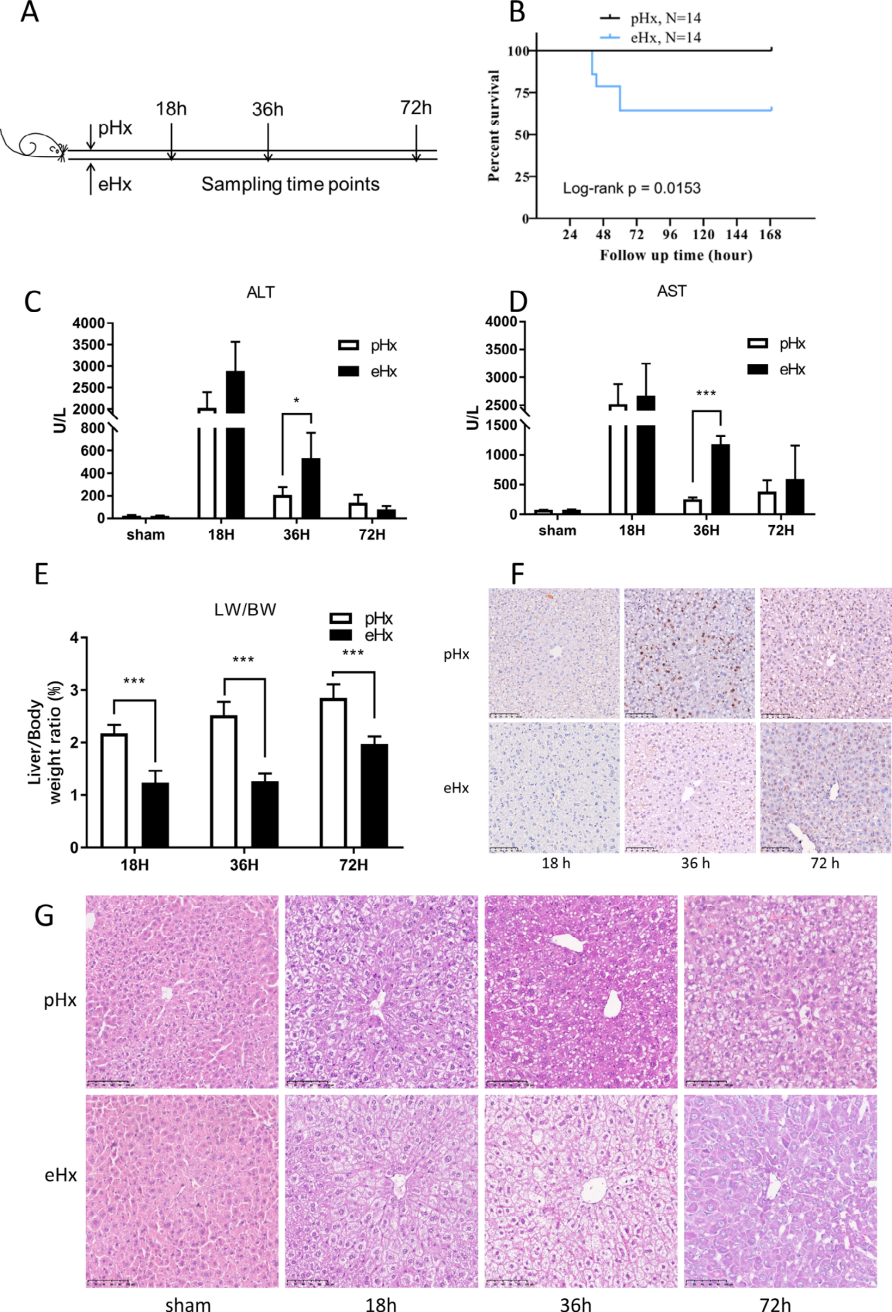
Construction and prognosis of pHx and pHx mice model. (**A**) Sampling time points after pHx and eHx. (**B**) Mice were subjected to hepatectomy and survival was monitored for 7 days (pHx [n = 14] and eHx [n = 14]). (**C, D**) Serum ALT and AST levels was detected in 18 h,36 and 72 h after pHx and pHx (n ≥ 3). (**E**) liver/body weight ratio was observed after pHx and pHx (n ≥ 3). (**F**) Ki-67 staining for liver sections of the mice liver samples at 18 h, 36 and 72 h after hepatectomy and sham.(**G**) H&E staining for liver sections of the mice liver samples at 18 h, 36 and 72 h after hepatectomy. Error bars represent standard deviation (SD). ns: p > 0.05, no significance; *: p < = 0.05; **: p < = 0.01; ***: p < 0.001

The liver regeneration process posthepatectomy is broadly divided into initiation, proliferation, and termination phases. The peak of DNA replication after pHx in mice always occurs approximately 36 h after the operation [7]. Accordingly, to include all three phases in the analysis [26], we collected liver tissues at 18, 36, and 72 h after pHx and eHx. Standard pHx and eHx were performed on mice (Fig. 1A). The survival rates of the mice after pHx and eHx were 100% and 64.29%, respectively (Fig. 1B). Serum biochemical indices and liver tissues were collected to evaluate liver injury. Compared with pHx, eHx was associated with higher serum ALT and AST levels, particularly at 36 h posthepatectomy (Fig. 1 C and D). We further characterized liver regeneration in our models. An increased liver weight to body weight ratio (LW/BW) was observed after surgery in both models, and the pHx group exhibited a better regeneration compared with the eHx group (Fig. 1E). This was further confirmed by staining with the cell proliferation marker, Ki67 (Fig. 1F). H&E staining showed that the hepatic lobule structure was complete, and the hepatocytes were neatly arranged in the sham group. However, the pHx and eHx groups showed hepatocytomegaly, cytoplasmic rarefaction, and a loss of visible hepatic cord structure 36 h after hepatectomy (Fig. 1G).

### Global transcriptome profile of the liver posthepatectomy

**Fig. 2:**
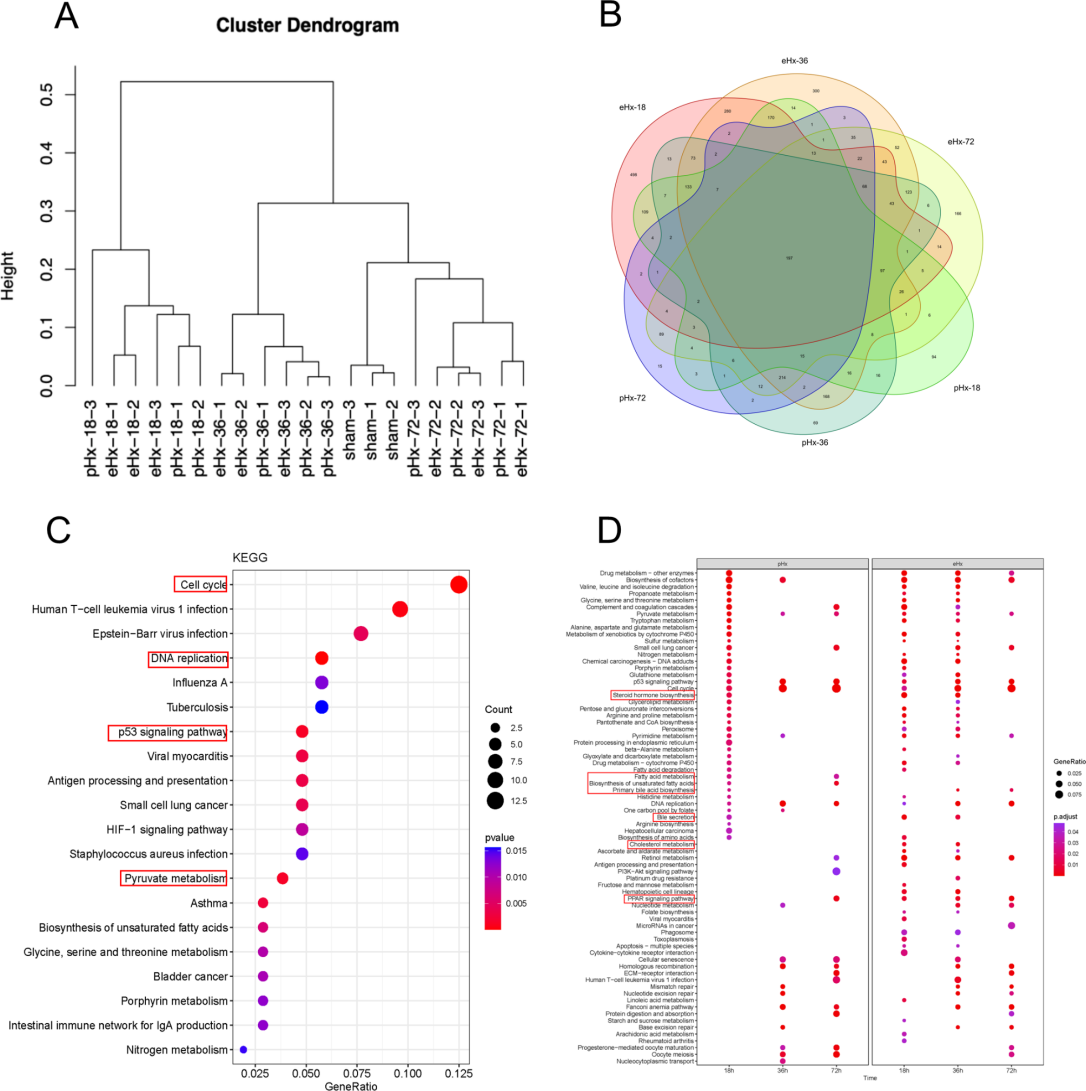
Comprehensive analysis of global transcriptome. (**A**) Cluster dendrogram of different samples. (**B**) Venn diagram of the number of DEGs in 6 pairwise comparisons: pHx-18 vs. sham, pHx-36 vs. sham, pHx-72 vs. sham, eHx-18 vs. sham, eHx-36 vs. sham, eHx-72 vs. sham. (**C**) KEGG enrichment of the common DEGs in 6 pairwise comparisons. (**D**) KEGG enrichment of DEGs in pHx vs. sham and eHx vs. sham at 18 h, 36 and 72 h time points

A total of 14,505 filtered genes were reserved for differential analysis (Table S1). Cluster analysis showed that the samples were grouped into the same group mainly based on differences in processing time, indicating that the changes in the transcriptome after hepatectomy changed over time. In addition, the samples before hepatectomy and 72 h post pHx were relatively similar (Fig. 2A), suggesting that the transcriptome of the samples closely returned to the initial state 72 h post pHx. The results of the difference analysis showed that at three different time points after surgery, the total number of differentially expressed genes after eHx was greater than that after pHx. At 18, 36, and 72 h posthepatectomy, there were 1842, 2129, and 1277 DEGs in the eHx group, respectively, when compared to the sham groups, while only 962, 1305, and 732 DEGs were found in the pHx group, respectively (Table 1). These findings indicate that eHx induced more drastic and extensive transcriptome changes than pHx. To further study the expression pattern of DEGs and explore differences between pHx and eHx, we counted upregulated and downregulated DEGs (Figure S1). A Venn diagram of DEGs was drawn in 6 pairwise comparisons: pHx-18 vs. sham, pHx-36 vs. sham, pHx-72 vs. sham, eHx-18 vs. sham, eHx-36 vs. sham, and eHx-72 vs. sham. (Fig. 2B), there are 197 common genes in the collection of all comparisons, suggested these genes were involved in liver injury and regeneration post-pHx and -eHx. KEGG enrichment was employed to analyse these common genes (Fig. 2C). The result showed that common enriched pathways after both pHx and eHx included DNA replication, p53 signaling pathway, cell cycle, pyruvate metabolism, DNA replication and cell cycle, which are important pathways for the whole process of liver regeneration. The energy and substrate required for cell regeneration may be provided by pyruvate metabolism. KEGG enrichment was carried out to analyse the DEGs of pHx vs. sham and eHx vs. sham at three different time points. The results showed that the two comparison groups differed at 18, 36, 72 h time points, and the most common pathways after both pHx and eHx are same to result of the 197 genes KEGG enrichment. In addition, we found some pathways associated with lipid metabolism were significantly different, including steroid hormone biosynthesis, fatty acid metabolism, biosynthesis of unsaturated fatty acids, primary bile acid biosynthesis, bile secretion, cholesterol metabolism, and PPAR signaling pathway (Fig. 2D), suggesting that these pathways related to lipid metabolism are related to liver regeneration after eHx.

**Table 1:** Total number of signature DEGs in different comparison

Contrast	Up	Down	Sum
pHx-18 vs. sham	419	543	962
pHx-36 vs. sham	828	477	1305
pHx-72 vs. sham	596	136	732
eHx-18 vs. sham	806	1036	1842
eHx-36 vs. sham	1172	957	2129
eHx-72 vs. sham	899	378	1277
pHx-36 vs. pHx-18	503	103	606
pHx-72 vs. pHx-18	507	145	652
eHx-36 vs. eHx-18	504	94	598
eHx-72 vs. eHx-18	1034	399	1433
pHx-72 vs. pHx-36	96	283	379
eHx-72 vs. eHx-36	320	183	503

Up: The number of up-regulated DEGs;Down: The number of down-regulated DEGs; Sum: summation

### Bioinformatic analysis of DEGs in eHx vs. pHx

**Fig. 3:**
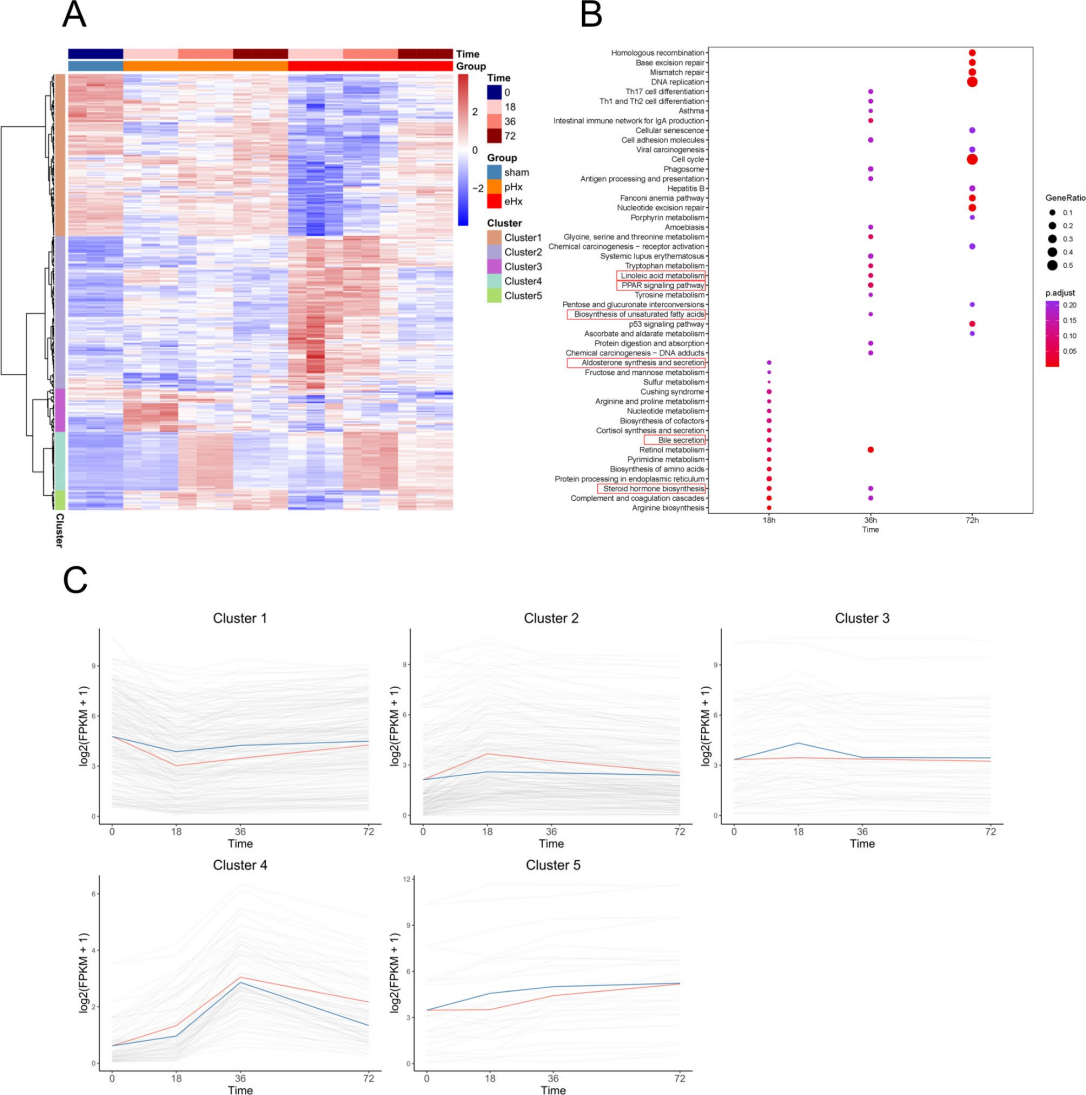
Analysis of DEGs of pHx and eHx in the transcriptome. (**A**) Heat map of DEGs of pHx and eHx. (**B**) KEGG enrichment of DEGs of pHx and eHx. (**C**) Trendgram of 5 clusters of DEGs in pHx and eHx

The expression patterns of all 14,505 reserved genes in eHx vs. pHx are shown in Table S2. Compared with pHx, the number of DEGs reached a maximum of 230 at 18 h after surgery, and the number of DEGs at 36 and 72 h decreased sequentially to 87 and 43 (Table 2), respectively, indicating that those DEGs may be involved in severe liver damage caused by eHx. Transcriptome changes in both pHx and eHx occurred during the early postoperative period, and the difference between the two groups decreased with postoperative recovery. All the DEGs from the three time points posthepatectomy between eHx vs. pHx were included for cluster analysis (Fig. 3A). KEGG analysis of those DEGs showed differences in several common pathways, included DNA replication and cell cycle. Meanwhile, multiple other metabolic pathways were enriched, including bile secretion, steroid hormone biosynthesis, biosynthesis of unsaturated fatty acids, aldosterone synthesis and secretion, linoleic acid metabolism, and PPAR signaling pathway(Fig. 3B). It is suggested that these metabolic pathways are involved in the delayed liver regeneration of eHx. According to the expression patterns of DEGs, they were mainly clustered into five categories (Fig. 3C). Cluster 1, Cluster 3, and Cluster 5 included the downregulated genes at 18 h (eHx vs. pHx). Cluster 2 included the upregulated genes at 18 h (eHx vs. pHx). Cluster 4 included the downregulated genes at 72 h. The top 10 DEGs of each cluster are listed in Figure S2. It is suggested that a large number of genes and pathways are involved in liver regeneration after liver resection.

**Table 2:** Total number of signature DEGs of eHx vs. pHx at different time pionts

Contrast	Up	Down	Sum
eHx-18 vs. pHx-18	103	127	230
eHx-36 vs. pHx-36	36	51	87
eHx-72 vs. pHx-72	39	4	43

Up: The number of up-regulated DEGs;Down: The number of down-regulated DEGs; Sum: summation

### Overview of metabolomic profiling variation in the liver posthepatectomy

**Fig. 4:**
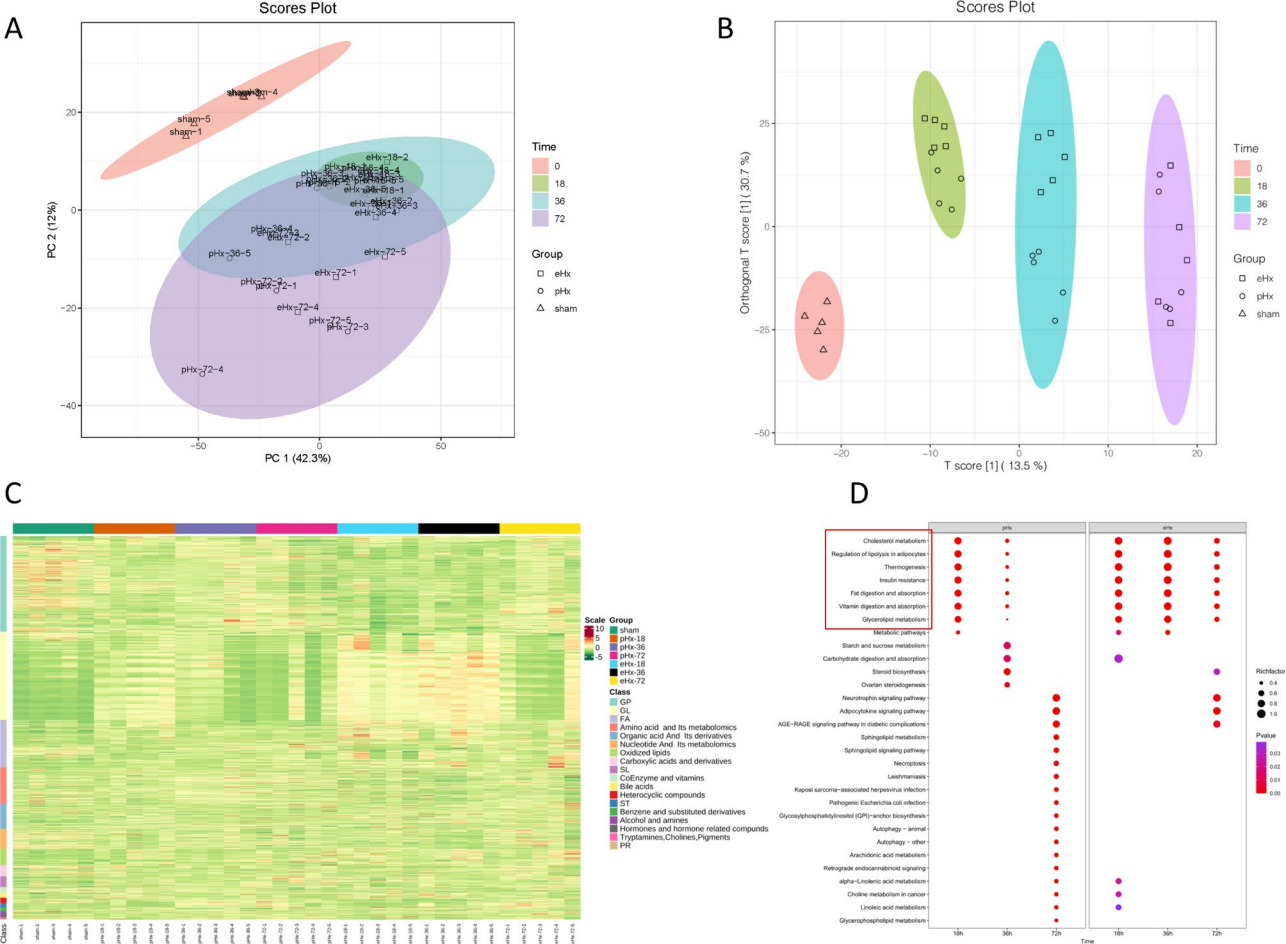
Landscape of the metabolic patterns of mouse liver after pHx and eHx treatments. (**A**) Principal component analysis on the metabolic profiles after pHx and eHx treatments; (**B**) OPLS-DA plot of mouse liver metabolomics; (**C**) Heat map displayed the relative level of metabolites after pHx and eHx treatments. The metabolites with high levels were shown in red, whereas the metabolites with low levels were shown in green. GP: glycerophospholipids, GL: glycerolipids, FA: fatty acyls, ST: sterol, SL: sphingolipids, PR: prenol lipids; (**D**) KEGG enrichment of DPMs in pHx vs. eHx at 18 h, 36 and 72 h time points

To further explore the influence of pHx and eHx on metabolic reprogramming within the liver, metabolomics analysis was performed on liver tissues at 18, 36, and 72 h after pHx and eHx. In total, 1383 metabolites were filtered (variable coefficient ≥ 0.3) in all groups (Table S3). The number of signature DPMs is shown in Table 3. In order to test the quality of metabolome data, PCA was conducted using the metabolic profiles of the different groups. PC1 for metabolomics explains 42.3% of the variance, and PC2 explains 12% of the variance using integral metabolomics data (Fig. 4A). An OPLS-DA plot was also generated to analyse mouse liver metabolomics (Fig. 4B). PCA and OPLS-DA plot showed that the metabolome data were acceptable and could be further analyzed. The metabolomic cluster dendrogram of different samples is shown in Figure S3. DPMs were grouped into several categories, including glycerophospholipids, glycerolipids, fatty acyls, sterols, amino acid and their metabolites, nucleotides and their metabolites. This suggests that liver regeneration requires not only the synthesis of nucleic acids and proteins but also many other types of metabolites, including glycerophospholipids, fatty acyls and hormones (Fig. 4C). KEGG enrichment showed that there were many different pathways of DPMs in the pHx vs. sham and eHx vs. sham comparisons at three different time points. Interestingly, seven pathways showed the same changing pattern, including cholesterol metabolism, regulation of lipolysis in adipocytes, thermogenesis, insulin resistance, fat digestion and absorption, vitamin digestion and absorption, and glycerolipid metabolism. The rich factor of the differential expression of these pathways was high at 18 h, decreased at 36 h, and disappeared at 72 h in the pHx groups. It was high at 18 and 36 h, and decreased at 72 h in the eHx groups. These findings imply these pathways are important for regeneration post both pHx and eHx, and compared to pHx, the process of regeneration of eHx was delayed (Fig. 4D). The results indicated that the metabolic patterns of both pHx and eHx mice at 18, 36, and 72 h were significantly different.

**Table 3:** Total number of signature DPMs identified in this study

groupName	totalSigMetabolites	Down-regulated	Up-regulated
Sham vs. pHx-18	509	145	364
Sham vs. pHx-36	310	79	231
Sham vs. pHx-72	399	184	215
Sham vs. eHx-18	632	224	408
Sham vs. eHx-36	592	166	426
Sham vs. eHx-72	471	114	357

groupName: Differentially expressed metabolites grouping information; totalSigMetabolites: The total number of DPMs; Up-regulated: The number of up-regulated DPMs; Down-regulated: The number of down-regulated DPMs.

### Bioinformatic analysis of DPMs in eHx vs. pHx

**Fig. 5:**
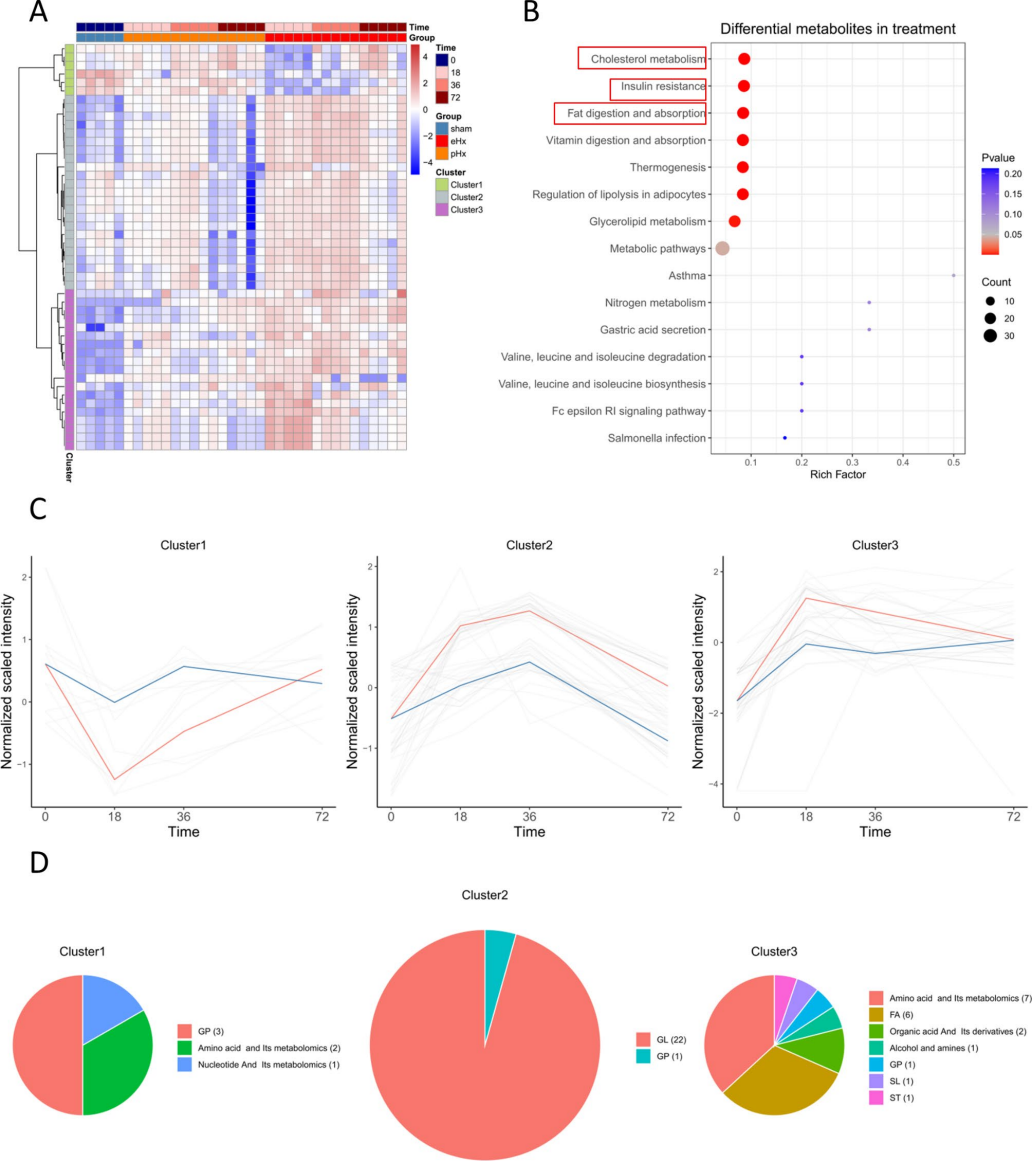
The analysis of DPMs between pHx and eHx. (**A**) The heatmap of significant differential expressed metabolites. (**B**) KEGG pathway analysis. (**C**) Trendgram of 3 clusters of DPMs in pHx and eHx. (**D**) Pie chart shows Metabolite composition of each cluster

Cluster analysis showed that these DPMs were grouped into three categories with different expression patterns (Fig. 5A). Pathway enrichment analysis was performed to analyse all of the DPMs. The results showed that the DPMs were enriched in several metabolic pathways, including cholesterol metabolism, insulin resistance, and fat digestion and absorption (Fig. 5B). The expression patterns of the three clusters were drawn. The DPMs of Cluster 1 reduced at 18 h, and then fell back at 36 h and 72 h. The DPMs of Cluster 2 increased at 18 h and 36 h, and then fell back at 72 h. The DPMs of Cluster 3 increased at 18 h, and then gradually fell back at 36 h and 72 h. These findings suggest that the process of liver regeneration requires the regulation of metabolite content, and the metabolite content gradually returned to normal levels at the late stage of liver regeneration (Fig. 5C). A pie chart showed the metabolite composition of each cluster (Fig. 5D). The TOP 5 DPMs were selected in clusters 1, 2, and 3 of pHx vs. eHx (Figure S4). The top 5 DPMs in Cluster 1 were glutathione reduced form, PI (20:4/16:0), 2’-deoxycytidine-5’-monophosphate, PS (20:2/20:0), and glutathione oxidized. In Cluster 3, the top 5 differently expressed metabolites included carnitine C8:1, allysine, 4-hydroxy-l-glutamic acid, histamine, and isobutyryl carnitine. The top 5 DPMs of Cluster 2 were five different triglycerides, implying that metabolic dysregulation of these DPMs may contribute to the failure of liver regeneration post-eHx.

### Integrative analysis of the metabolome and transcriptome

**Fig. 6:**
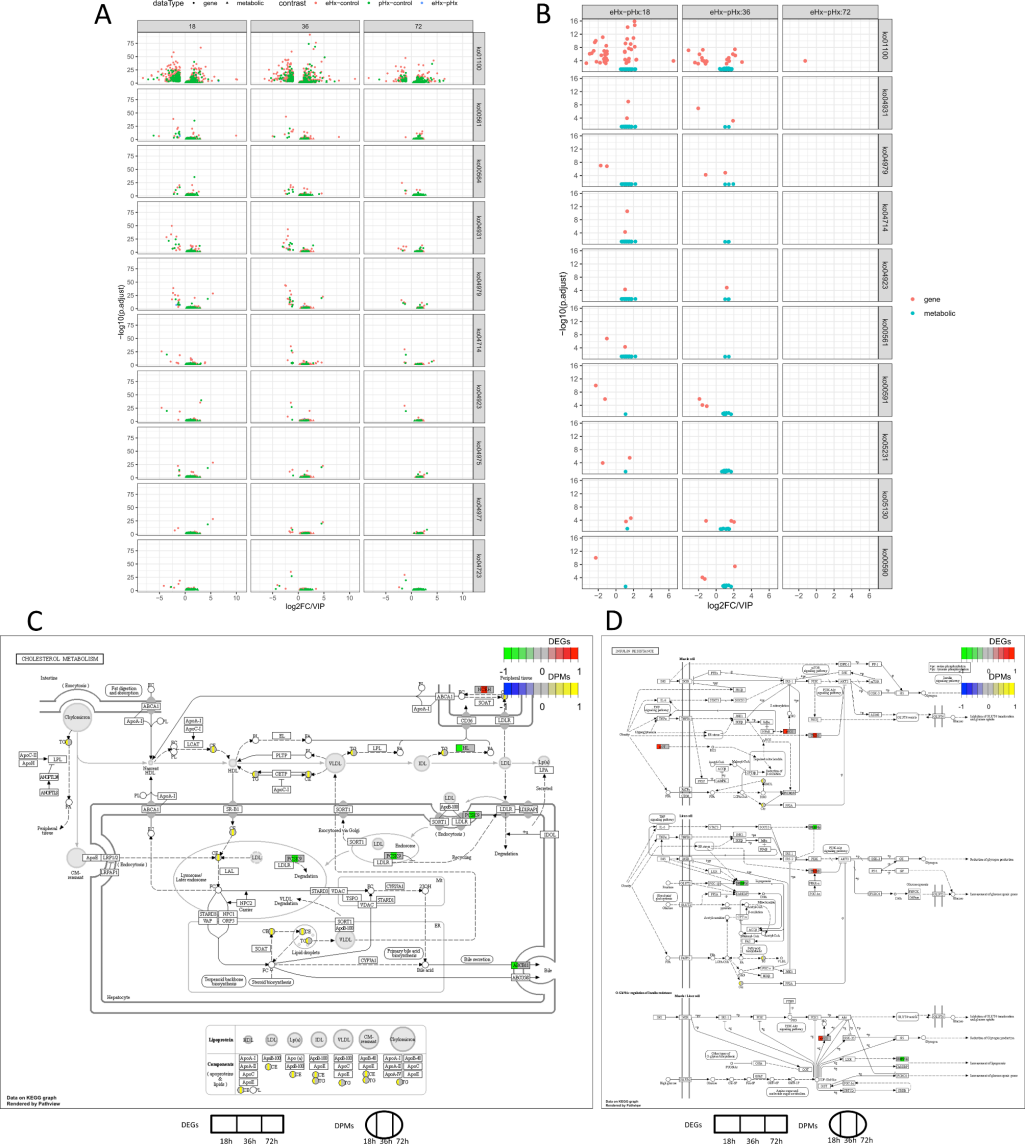
The integrated analysis of transcriptomics and metabolomics. (**A**) Composite chart of DEGs and DPMs of In pHx vs. sham and eHx vs. sham simultaneously enriched into same pathways. (**B**) Composite chart of DEGs and DPMs of In pHx vs. eHx simultaneously enriched into same pathways. (**C**) DEGs and DPMs in cholesterol metabolism. (**D**) DEGs and DPMs in insulin resistance

Integrative analysis of the DEGs and DPMs was performed to better understand the potential crosstalk between the transcriptome and metabolome after hepatectomy. Comparing the metabolomic and transcriptomic data, we built a composite chart of DEGs and DPMs, which were simultaneously enriched in the same pathways. Several pathways were identified in the pHx vs. sham and eHx vs. sham comparisons, including the metabolic pathway (ko01100), glycerolipid metabolism (ko00561), glycerophospholipid metabolism (ko00564), insulin resistance (ko04931), and cholesterol metabolism (ko04979) (Fig. 6A). To further explore differences between pHx and eHx, we built a composite chart of DEGs and DPMs in eHx vs. pHx, and found several important pathways, including the metabolic pathway, insulin resistance, cholesterol metabolism, thermogenesis (ko04714), and the regulation of lipolysis in adipocytes (ko04923) (Fig. 6B). Both comparisons contained the metabolic pathway, insulin resistance, and cholesterol metabolism. Because the metabolic pathway is a global metabolic pathway, we were interested in the insulin resistance and cholesterol metabolism pathways.

Importantly, the liver is the most important organ in cholesterol metabolism; thus, we integrated the metabolome and transcriptome data on the cholesterol metabolism pathways, which included DEGs and DPMs at three different sampling time points (Fig. 6C). The results of the integrative analysis supported the notion that cholesterol metabolism may play an important role in liver injury or regeneration after pHx and eHx. We also analysed the DEGs and DPMs involved in the insulin resistance pathway (Fig. 6D). The integrated analysis of transcriptomics and metabolomics provided potential implications for understanding the systemic mechanism of liver injury and regeneration after pHx and eHx.

### qPCR validation of selected genes

**Fig. 7:**
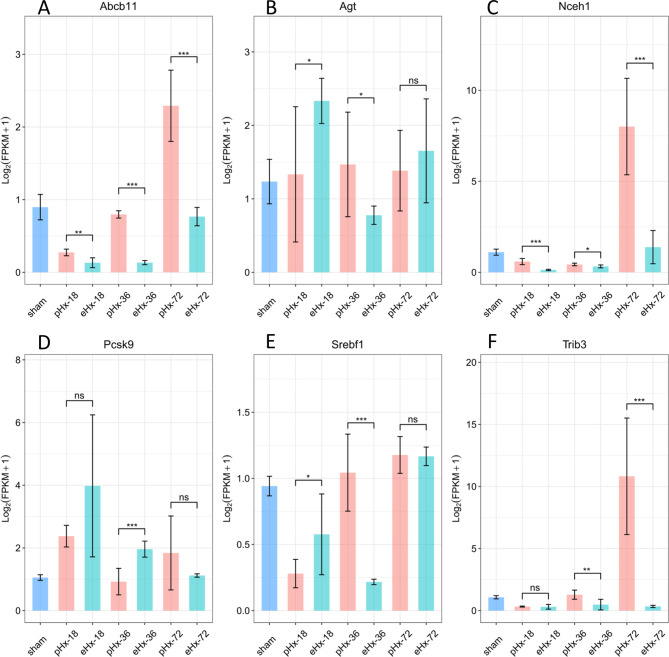
Expression level verification by qRT-PCR. (**A–F**) The expression level of Agt, Trib3, Srebf1, Abcb11, Nceh1, and Pcsk9. The average expression levels in the controls were set to 1. Error bars represent SD. ns: p > 0.05, no significance; *: p < = 0.05; **: p < = 0.01; ***: p < 0.001

Insulin resistance and cholesterol metabolism may play an important role in the liver injury or regeneration post-pHx and -eHx. The *Abcb11*, *Nceh1*, and *Pcsk9* genes of the cholesterol pathway and the *Agt*, *Srebf1*, and *Trib3* genes of the insulin resistance pathway were selected for qRT-PCR verification. A total of six DEGs were measured by qRT-PCR (Fig. 7). The expression trends of these DEGs were similar to those of RNA-seq (Figure S5 and S6), indicating that the RNA-Seq data were reliable. The result further illustrated the role of these genes in regeneration after hepatectomy.

## Discussion

### Selection of sample collection time point

We sampled mouse hepatic tissue at 18, 36, and 72 h posthepatectomy to capture the different stages of liver regeneration at the molecular level. According to the time kinetics of DNA synthesis in different liver cell types during liver regeneration after partial hepatectomy, the first peak of DNA synthesis in hepatocytes occurs at 18–24 h, with a smaller peak occurring at 36–48 h. The peak of DNA synthesis in biliary ductular cells and Kupffer cells is at approximately 36 h. Proliferative endothelial cells are identified from approximately 72 h post-pHx [27]. After 48–72 h, all cellular elements of the liver proliferate. The liver histology at 72–96 h after pHx is characterized by clumps of small hepatocytes surrounding capillaries [28]. Although we collected data at several time points, our study still lacked the appropriate time points to study metabolic changes involved in regeneration posthepatectomy. As such, further studies are needed to explore the transcriptome changes after extended hepatectomy. Transcriptomics revealed that the differential expression of genes between hepatectomy and expanded hepatectomy was significantly different at 18 and 36 h, and was closer to Sham at 72 h (Fig. 2A). However, the metabolomic data did not show that pHx and eHx were similar to Sham at 72 h (Figure S3). This suggested that DNA activity gradually returned to a normal state at 72 h posthepatectomy, but the metabolite synthesis involved in the procedure was still active.

### Similarities and differences in pHx and eHx transcriptome data

The process of liver regeneration posthepatectomy is accompanied by a large number of gene and pathway expression changes, and transcriptome analysis has been widely used in liver regeneration research [29, 30]. Ensuring the progression and completion of the cell cycle could facilitate liver regeneration and prevent the occurrence of SFSS [17]. Our research also emphasized that the cell cycle was important for all three phases of posthepatectomy liver regeneration. Pyruvate metabolism and DNA replication may be involved in progression. We observed that the Fanconi anemia pathway was significantly enriched at the 36 and 72 h time points in both pHx and eHx, and this pathway is important in hepatocyte gene repair [31]. Similarly, retinol metabolism could be involved in 72 h time point, and this process enhances posthepatectomy liver regeneration [32, 33]. Moreover, the ECM − receptor interaction could play the reverse role by regulating proper termination of liver regeneration [34].

We observed that progesterone − mediated oocyte maturation, oocyte meiosis and nucleocytoplasmic transport pathways may be involved in the S phase of the cell cycle of pHx at 36 h. Phagosome, PPAR signalling pathway, folate biosynthesis and several metabolic pathways may be the reason for the delayed liver regeneration after eHx. Many metabolism-related pathways were significantly enriched at the 18 and 36 h time points in eHx; these pathways included arachidonic acid metabolism, starch and sucrose metabolism, linoleic acid metabolism, fructose and mannose metabolism, retinol metabolism, cholesterol metabolism. The observed overload of metabolic pathway activities after eHx may be a major cause of liver failure, which was consist with a previous report [24].

In addition, some studies have shown that the Hedgehog pathway and hypoxia pathway participate in liver regeneration for both pHx and eHx [24]. The IL10 Pathway and the cAMP pathway may regulate hepatocyte proliferation after pHx [35, 36]. However, similar results were not found in this study, which may be attributed to experimental design and sample individual differences.

### Metabolomic analysis of pHx and eHx

It has been reported that liver metabolites remarkably change after pHx [37]. Glucose metabolism, lipid metabolism, amino acid metabolism, and bile acid metabolism, are essential pathways for liver regeneration [38, 39]. We also found a significant change in amino acid metabolism and bile acid metabolism during regeneration post pHx and eHx (Figs. 4C and 5D). In addition, we observed that DPMs were enriched in glycerophospholipids, glycerolipids, and fatty acid. Plasma glycerophospholipids are associated with liver regeneration [40]. Yang et al. (2020) found that glycerolipid metabolism regulates axonal growth and regeneration [41], but the function of glycerolipids in liver regeneration is still unclear. There were several distinct DPM-enriched pathways, such as autophagy, that appeared at 72 h post pHx (Fig. 4D), and these pathways might play a role in balancing the regulation of cell division and apoptosis in the termination phase of liver regeneration post pHx. These pathways did not appear at 72 h post eHx, implying that the termination phase of liver regeneration was delayed in eHx mice. By analysing the DPMs between eHx and pHx, we found that significant DPMs were enriched in cholesterol metabolism, thermogenesis, insulin resistance, fat digestion and absorption, and vitamin digestion and absorption pathways (Fig. 5B). These were the same seven pathways that showed the same changing pattern in Fig. 4D, implying that these metabolic pathways might be involved in liver regeneration in eHx. The changes in the content of these metabolites may be caused by insufficient liver metabolic function caused by the reduction in liver volume after liver resection, or by the need for liver regeneration. The specific functional mechanisms need further research and exploration. Our objective was to gain insight into the disparity between eHx and conventional pHx in terms of metabolism, and to investigate the potential mechanisms underlying injury and regeneration through a collaborative analysis. Our work has filled the knowledge gap regarding injury and regeneration after hepatectomy.

### Cholesterol metabolism and insulin resistance pathways may be involved in liver injury and regeneration after hepatectomy

To explore the correlation between the transcriptome and metabolome posthepatectomy, we performed an integrated analysis of transcriptomics and metabolomics to explore the transcriptional and metabolic changes after hepatectomy. Among the significantly changed genes and metabolites, we focused on several pathways, including insulin resistance and cholesterol metabolism (Fig. 6A and B). These pathways were enriched by both DEGs and DPMs, which suggests that these pathways are involved in liver regeneration posthepatectomy on both the transcriptional and metabolic levels. The liver is the central organ for lipogenesis, gluconeogenesis, and cholesterol metabolism [42]. Cholesterol is an essential structural component of cellular membranes. The majority of cholesterol is embedded within the lipid bilayer, and some members serve as precursors for several signalling pathways [43]. The enzymes of cholesterol biosynthesis are changed during liver regeneration, and cholesterol has an active role in cell proliferation and liver regeneration [44]. Cholesterol induces hepatocyte proliferation and liver regeneration in mouse models of NASH and fibrosis [45]. A high-cholesterol diet and pravastatin sodium have both been shown to influence the initiation of liver regeneration in rat post-partial hepatectomy [46]. In this study, we found that cholesterol metabolism was disturbed after pHx and eHx, and this result is consistent with that of previous reports. Additionally, DEGs and DPMs between eHx and pHx were enriched in cholesterol metabolism in eHx vs. pHx, which implies that cholesterol metabolism is involved in liver injury and even liver failure after eHx. Insulin resistance is another DEG- and DPM-enriched pathway identified in this study. Insulin resistance is associated with numerous metabolic disorders [47] and is a common phenomenon in mice after hepatectomy. Insulin resistance is consistent with the clinical features of metabolic disorders, and the clinical outcome is negatively correlated with postoperative insulin resistance [48]. In this study, the enrichment of DEGs and DPMs in insulin resistance implies that insulin resistance plays important roles in liver injury and regeneration after eHx and pHx, but the exact mechanisms underlying the development of postoperative insulin resistance are not clear. Our findings further confirm that cholesterol metabolism and insulin resistance may be involved in liver injury and regeneration after pHx and eHx. Although many studies have reported correlations between these two pathways and liver regeneration or injury, many details still need further study.

## Conclusions

In this study, an integrative analysis of the transcriptome and metabolome of the mouse liver tissues post-pHx and eHx was used to assess differences between the two procedures. Based on differences in the transcriptional and metabolic profile of the pHx and eHx models, dysregulated cholesterol metabolism and insulin resistance pathways were closely correlated to liver failure and individual death post-eHx. Moreover, the comparison between pHx and eHx models provided a deeper understanding of liver injury and regeneration posthepatectomy.

## Methods

### Animals

Adult male C57BL/6J (25–30 g, approximately 8 weeks old) mice were housed (≤ 5 per cage) in a temperature- and humidity-controlled environment on a 12:12 h light–dark cycle with access to food and water ad libitum. All experiments involving mice were approved by the Laboratory Animal Care and Use Committee of Guangxi Medical University and performed in accordance with the National Guidelines for the Care and Use of Animals. Mice recruited in the trial were divided stochastically into two batches. The first batch of mice was used for survival rate, and recorded from Days 0 to 7 (14 mice per group). The second batch of mice was randomly divided into three groups (8 mice per time point for each group): the sham group, the pHx group and the eHx group. The research workflow is given in the supplementary material (Figure S7). C57Bl/6 mice were obtained from Laboratory animal centre of Guangxi Medical University (Guangxi, China).

### Hepatectomy

Hepatectomy was performed under inhalational anesthesia with 3% isoflurane for induction and 1.5% isoflurane for maintenance (R510-22-10, Shenzhen RWD Life Technology Co., Ltd). pHx was performed as previously described [49], with resection of the median and left lateral liver lobes. eHx was performed as previously described [17], with the removal of the medial, left lateral, caudate, and inferior portion of the right lobes. All of the mice were kept on a heating pad following hepatectomy. After the operation, all mice were euthanized at 18, 36, or 72 h (the sham group was euthanized at 0 h) before liver tissue was harvested. Then, we collected serum to be centrifuged and placed fresh liver tissue in a liquid nitrogen tank for preservation.

### RNA extraction and qRT-PCR

Total RNA was extracted from mouse liver tissue samples using RNAiso Plus (#9109, Takara, Japan), and 500 ng of the isolated RNA was reverse transcribed to cDNA using MonScript™ RT Mix (#MR05101, Monad, China). The expression of genes was quantified using MonAmp™ qPCR Mix (#MQ00501S, Monad, China) on a CFX96™ Real-Time-System (Bio-Rad, USA). The primers are shown in Table S4.

### Hematoxylin-eosin (HE) staining

Liver samples were fixed with 4% paraformaldehyde for 48 h, and processed routinely for paraffin-embedded sections. HE staining was conducted with an HE Staining Kit (G1120, Solarbio, China) according to the manufacturers’ instructions. Briefly, paraffin sections were deparaffinized, hydrated, stained, dehydrated and sealed. Then, the sections were observed under a microscope and photographed.

### Immunohistochemical analysis (IHC)

Following antigen retrieval, the paraffin sections were blocked in 5% BSA for 1 h, incubated with specific primary antibodies againt Ki-67(1:2000 dilution, ProteinTech, USA) at 4 °C for overnight, and then incubated with a secondary antibody kit (PV-9001, ZSGB-BIO, China). After washing, the sections were stained with DAB (ZLI-9019, ZSGB-BIO, China) and counterstained with hematoxylin. The sections were observed under a microscope and analysed using ImageJ software.

### Extraction of hydrophilic compounds

All samples were thawed on ice. Each sample (50 ± 2 mg) was added to cold steel balls and homogenized at 30 Hz for 3 min. Then 1 mL of 70% methanol was added with internal standard extract to the homogenized centrifuge tube and mixed for 5 min. Then the samples were centrifuged at 12,000 rpm at 4 °C for 10 min. The supernatant (approximately 400 µl) was placed into a new EP tube and stored at − 20 °C. The samples were then centrifuged at 12,000 rpm at 4 °C for 3 min, and then the supernatants were aliquoted into corresponding bottles for analysis.

### Extraction of hydrophobic compounds

All samples were thawed on ice, and the samples (20 mg) were homogenized in a 1-mL mixture (methanol, methyl tert-butyl ether, and internal standard mixture) with cold steel balls (precooled on ice). The steel balls were removed, and the samples were mixed for 15 min. Then water (200 µl) was added, the samples were mixed for 1 min, and centrifuged at 12,000 rpm at 4 ºC for 10 min. Then 300 µl of supernatant was pipetted, and the supernatant was dried to a powder. The powder was dissolved with 200 µl mobile phase B solution and stored at − 80 ºC. Finally, the dissolving solution was injected into the sample bottle for analysis.

### LC-ESI-MS/MS analysis of hydrophilic metabolites and hydrophobic compounds

The samples were analyzed using an LC-ESI-MS/MS system (UPLC, ExionLC AD) as previously described with modification [50]. The analytical conditions were as follows, (1) UPLC: column, Waters ACQUITY UPLC HSS T3 C18; (2) column temperature: 40 °C; (3) flow rate: 0.4 mL/min; (4) injection volume: 2 µL; (5) solvent system: water (0.1% formic acid): acetonitrile (0.1% formic acid); and (6) gradient program: 95:5 (V/V) from 0 to 11.0 min, 10:90 (V/V) from 11.0 to 12.0 min, 10:90 (V/V) from 12.0 to 12.1 min, 95:5 (V/V) from 12.1 to 14.0 min, and 95:5 (V/V) at 14.0 min.The hydrophobic samples were analyzed using an LC-ESI-MS/MS system (UPLC, ExionLC AD) as previously described with modifications [51]. The analytical conditions were as follows: (1) column: Thermo Accucore™ C30; (2) solvent, A: acetonitrile/water (60/40, V/V, 0.1% formic acid, 10 mmol/L ammonium formate), B: acetonitrile/isopropanol (10/90 V/V, 0.1% formic acid, 10 mmol/L ammonium formate); (3) gradient program, A/B (80:20, V/V) at 0 min, 70:30 V/V at 2.0 min, 40:60 V/V at 4 min, 15:85 V/V at 9 min, 10:90 V/V at 14 min, 5:95 V/V at 15.5 min, 5:95 V/V at 17.3 min, 80:20 V/V at 17.3 min, and 80:20 V/V at 20 min; (4) flow rate, 0.35 ml/min; (5) temperature, 45 ºC; and (6) injection volume: 2 µl.

### RNA-seq and gene expression analysis

A total amount of 1 µg RNA per sample was used as RNA sample. Oligo(dT) magnetic beads were used to enrich mRNA with polyA structure in total RNA, and the RNA was broken into fragments of approximately 300 bp in length by ion interruption. Using the RNA as a template to synthesize double-stranded DNA, PCR amplification was used to enrich the library fragments. Then, the library was inspected by an Agilent 2100 Bioanalyzer (Agilent Technologies, Palo Alto, Calif, USA.), and the total concentration and effective concentration of the library were detected. The hybrid library was uniformly diluted to 2 nM. The library preparations were sequenced on an Illumina novaseq 6000 platform, and 125 bp/150 bp paired-end reads were generated. Raw sequencing read data were subjected to quality control analysis by FastQC. Reads were mapped to the mouse reference genome (GRCm38.p6) [52]. The genes in all groups with a mean expression of < 3 read counts were filtered out. The varFilter method of the R package geneFilter (V1.74.1) was used to screen the top 50% of the genes with variance values, and then the samples were clustered based on Pearson correlation. Differentially expressed genes were selected by DESeq2 (V1.32.0) [53] based on padj < 0.001 & |log2FC| > 1 [53]. ClusterProfiler was used for enrichment analysis, including GO enrichment and KEGG enrichment analyses [54–56].

### Metabolomics analysis

Five replicates from each group were collected for metabolomics analysis based on a high-resolution LC-MS platform. Based on the quality control sample, low replicates were removed with a threshold of CV < 30% and scaled by Pareto scaling [57]. Significant differentially produced metabolites were selected by OPLS-DA analysis with MetaboAnalystR (V3.1.0) [58]. Cluster heatmap analysis was performed on all samples, and a cluster heatmap was drawn by R package pheatmap (V1.0.12) (https://CRAN.R-project.org/package=pheatmap). The Student’s *t* test method was used to calculate the difference between pHx and eHx groups at each time point based on the metabolite standardized expression data, and the MULTIPLE test *P* values were corrected by the FDR method. Metabolites with FDR < 0.05 and VIP ≥ 1.5 were selected as significant differentially produced metabolites (DPMs).

### Integrated network analysis of the transcriptome and metabolome

The cojoint analyses of DEGs and DPMs were conducted by the cor command in R (v3.5.0) [59] (genemetabolite networks with a Pearson correlation coefficient (PCC) > 0.8 were used to construct the transcript-metabolite network). According to the results of the association analysis between DEGs and DPMs, an association network diagram was drawn. The DEGs and DPMs pathways were analyzed, and their common pathway information was mapped to KEGG [54].

### Statistics

Data were expressed as the mean ± SD. Significant differences between groups were determined by an ANOVA, with a Bonferroni correction for continuous variable and multiple groups. A two-tailed Student’s *t* test was used for the comparison of normally distributed continuous variables between two groups. For the survival studies, Kaplan–Meier log-rank analysis was performed. *P* values < 0.05 were considered statistically significant.

### Electronic supplementary material

Below is the link to the electronic supplementary material.

**Additional file 1: Table S1.** All the reserved genes for differential analysis, the genes in all groups with a mean expression of < 3 read counts were filtered out.

**Additional file 2: Figure S1.** The overall distribution of up and down regulated DEGs.

**Additional file 3: Table S2.** Expression patterns of all 14,505 reserved genes in eHx vs. pHx.

**Additional file 4: Figure S2.** The expression pattern of TOP10 DEGs of 5 clusters of pHx and eHx.

**Additional file 5: Table S3.** 1383 metabolites were filtered (variable coefficient ≥ 0.3) in all groups.

**Additional file 6: Figure S3.** Metabolomic cluster dendrogram of different samples.

**Additional file 7: Figure S4.** The TOP 5 DPMs were selected in clusters 1, 2, and 3 of pHx vs. eHx.

**Additional file 8: Figure S5.** The expression trends of selected DEGs of RNA-seq.

**Additional file 9: Figure S6.** The pearson correlation analysis between qRT-PCR and RNA-seq.

**Additional file 10: Figure S7.** The experimental workflow chart of this study.

**Additional file 11: Table S4.** Primers for DEGs q-PCR analysis.

## Data Availability

All data is available in the manuscript or the supplementary materials. All raw high-throughput data is deposited in NCBI Sequence Read Archive (SRA) database with the link of https://www.ncbi.nlm.nih.gov/bioproject/PRJNA960829. The bioProject accession is PRJNA960829.All application codes were uploaded in GitHub (https://github.com/Isivel/Transcriptome-and-metabolome-analysis-of-pHx-eHx).

## List of Abbreviations

pHx: partial hepatectomy
eHx: extended hepatectomy
SFSS: Small for Size Syndrome
HE: staining hematoxylin-eosin staining
DEGs: differentially expressed genes
DPMs: differentially produced metabolites
PLS-DA: partial least square-determinant analysis
PCA: principal component analysis
VIP: variable importance to projection
