# Supplementary figures and images for: Transcriptome sequencing and metabolome analysis reveal the metabolic reprogramming of partial hepatectomy and extended hepatectomy
Source: BMC Genomics. 2023 Sep 7;24:532. doi: 10.1186/s12864-023-09647-0 (PMC10486020; doi:10.1186/s12864-023-09647-0)

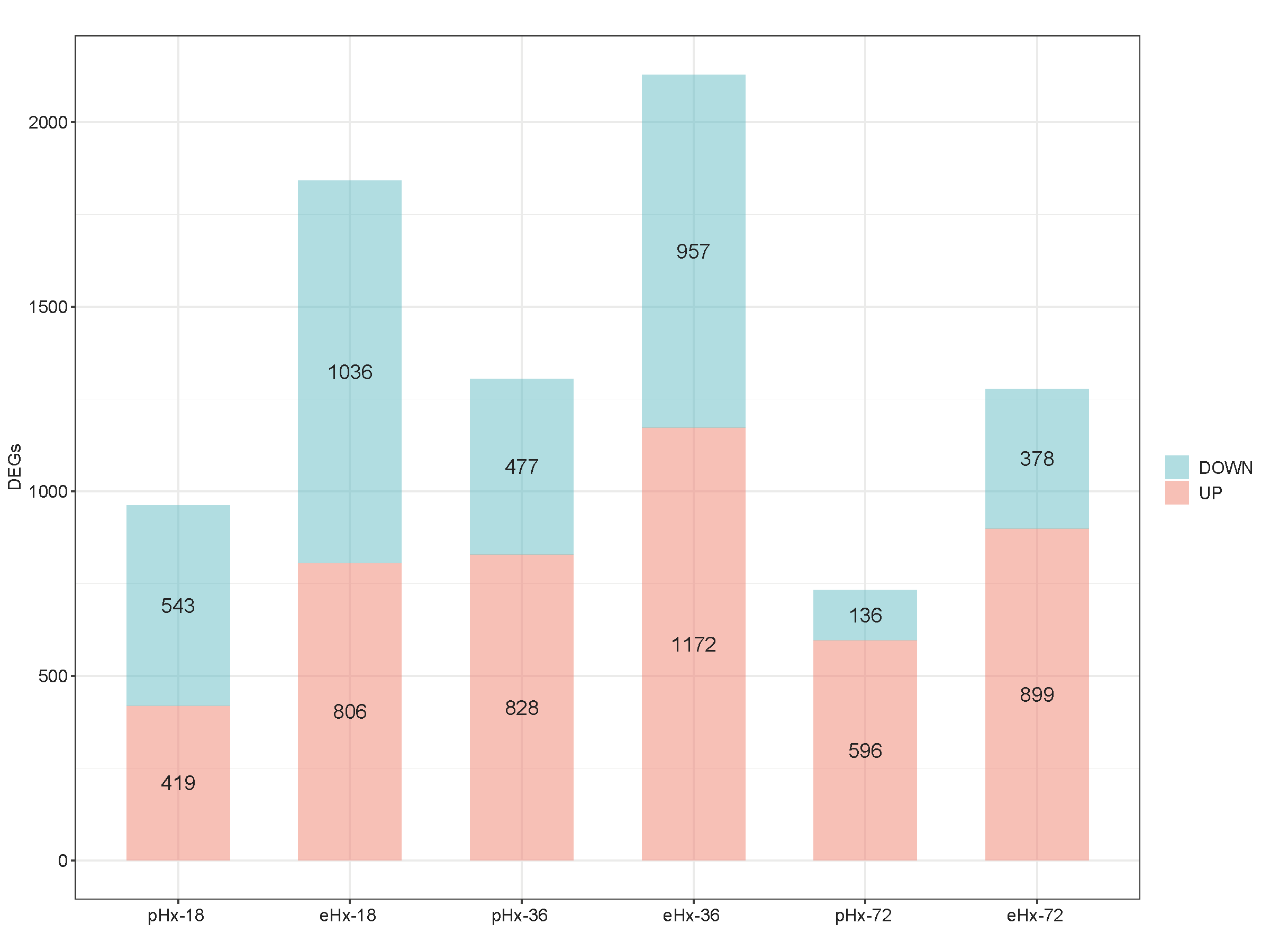

Supplement: Supplementary file 2 — Additional file 2: Figure S1. The overall distribution of up and down regulated DEGs. [file 12864_2023_9647_MOESM2_ESM.png]

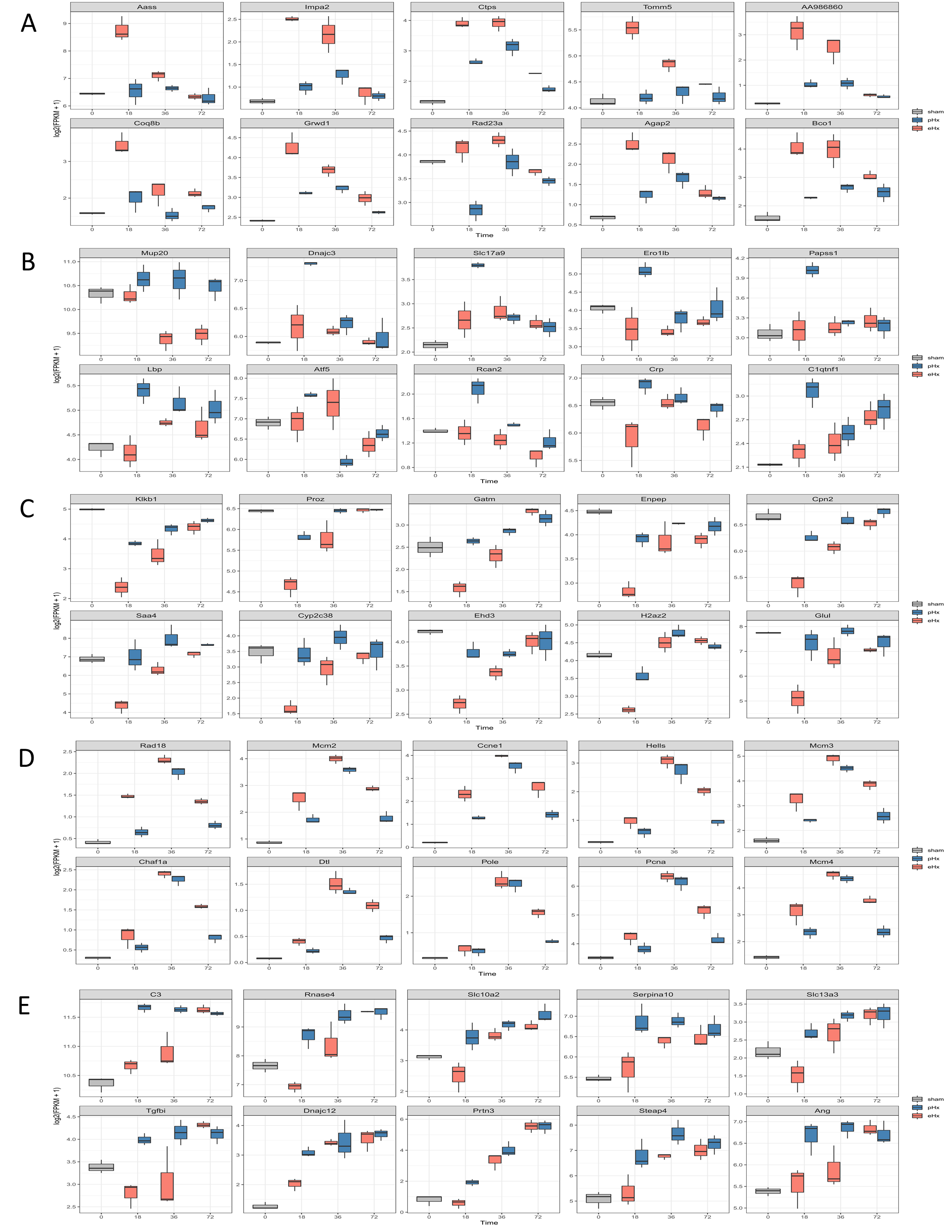

Supplement: Supplementary file 4 — Additional file 4: Figure S2. The expression pattern of TOP10 DEGs of 5 clusters of pHx and eHx. [file 12864_2023_9647_MOESM4_ESM.png]

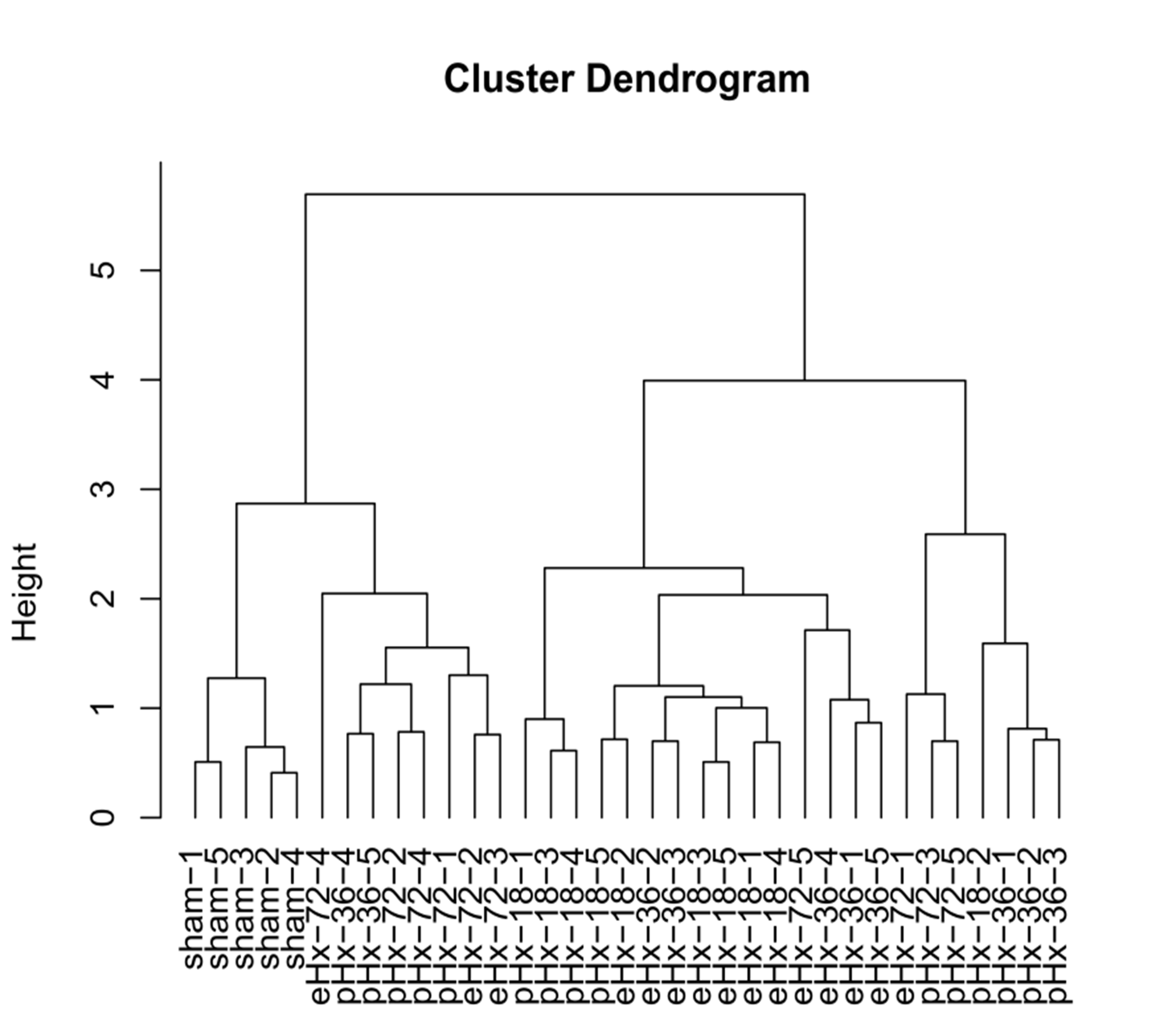

Supplement: Supplementary file 6 — Additional file 6: Figure S3. Metabolomic cluster dendrogram of different samples. [file 12864_2023_9647_MOESM6_ESM.png]

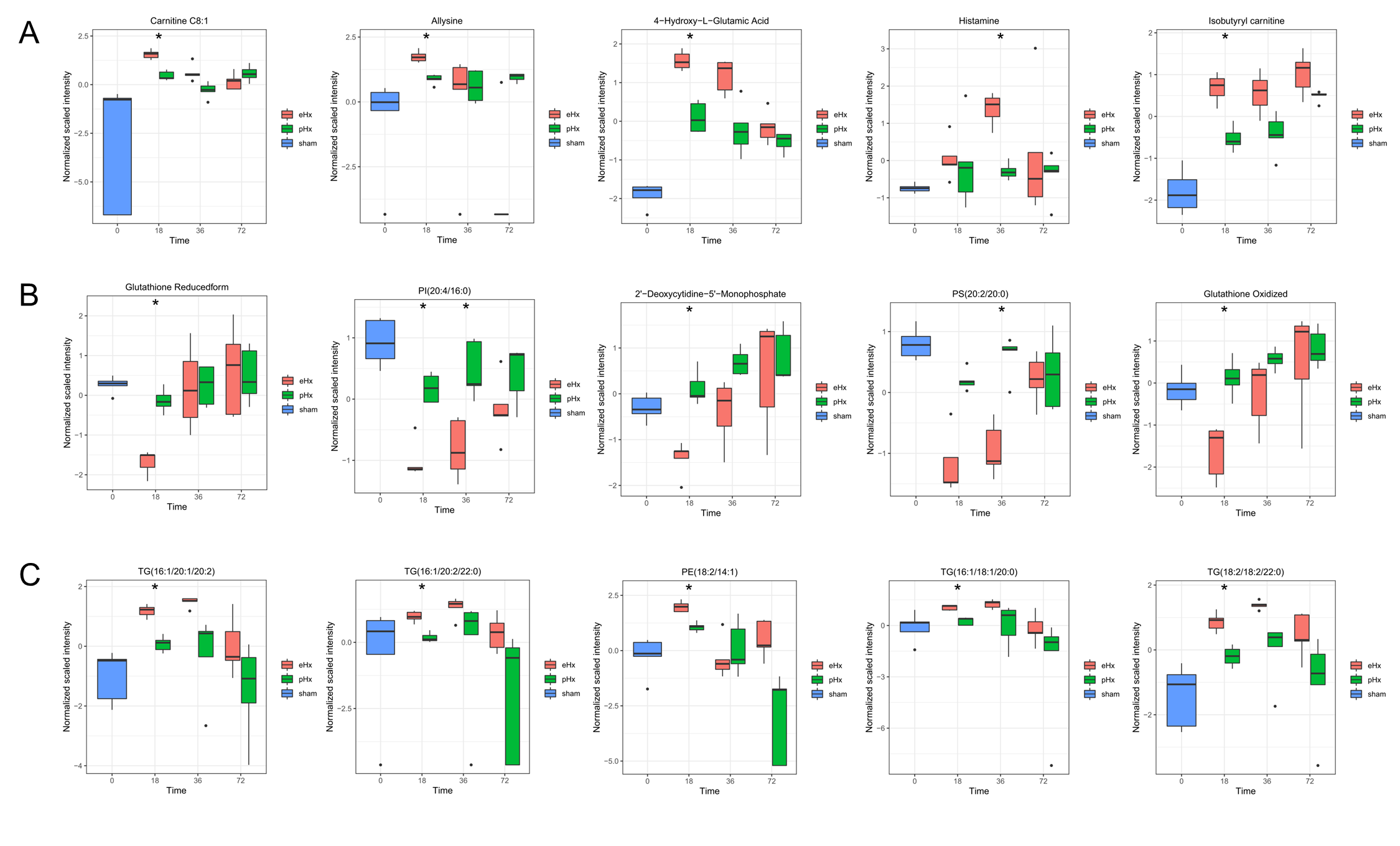

Supplement: Supplementary file 7 — Additional file 7: Figure S4. The TOP 5 DPMs were selected in clusters 1, 2, and 3 of pHx vs. eHx. [file 12864_2023_9647_MOESM7_ESM.png]

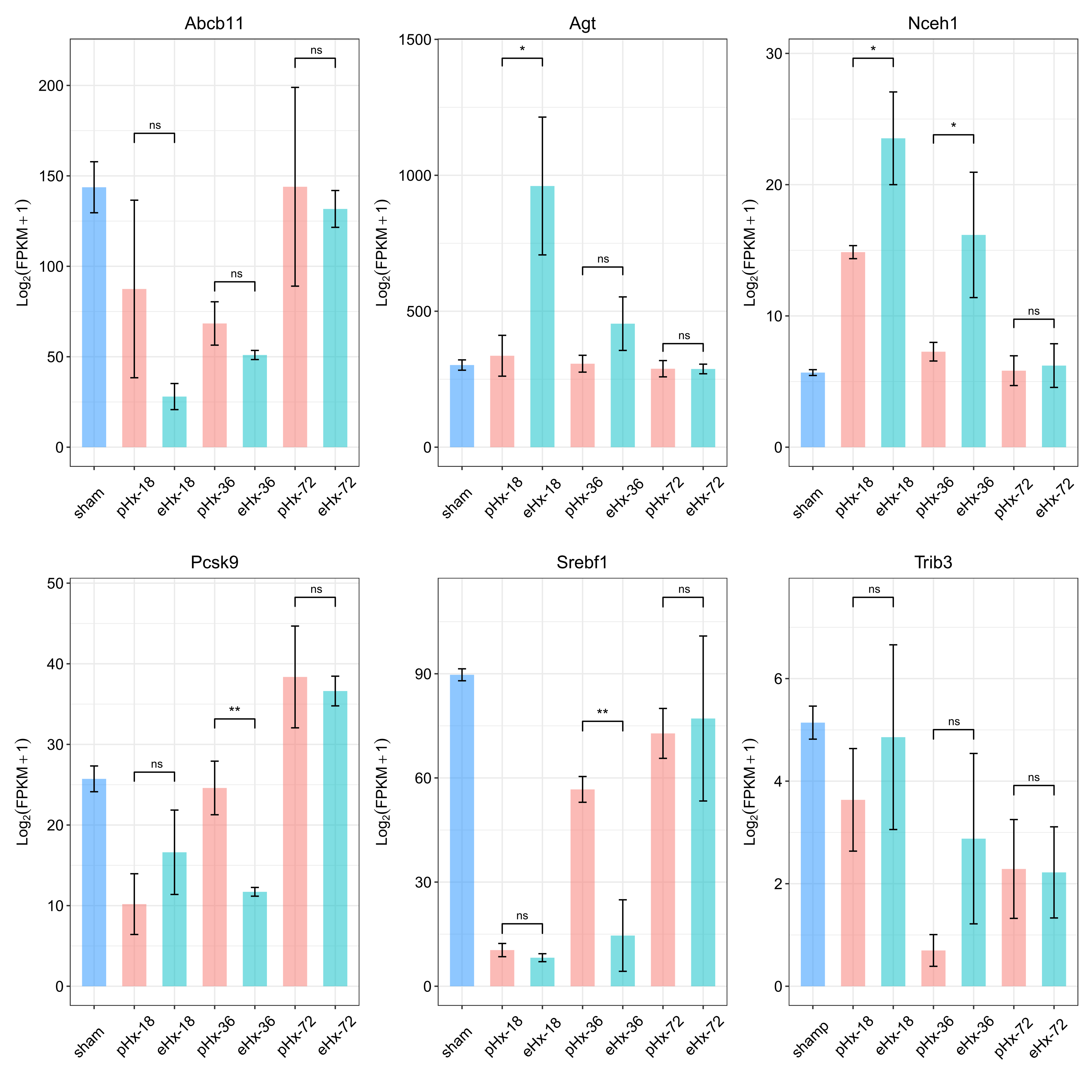

Supplement: Supplementary file 8 — Additional file 8: Figure S5. The expression trends of selected DEGs of RNA-seq. [file 12864_2023_9647_MOESM8_ESM.png]

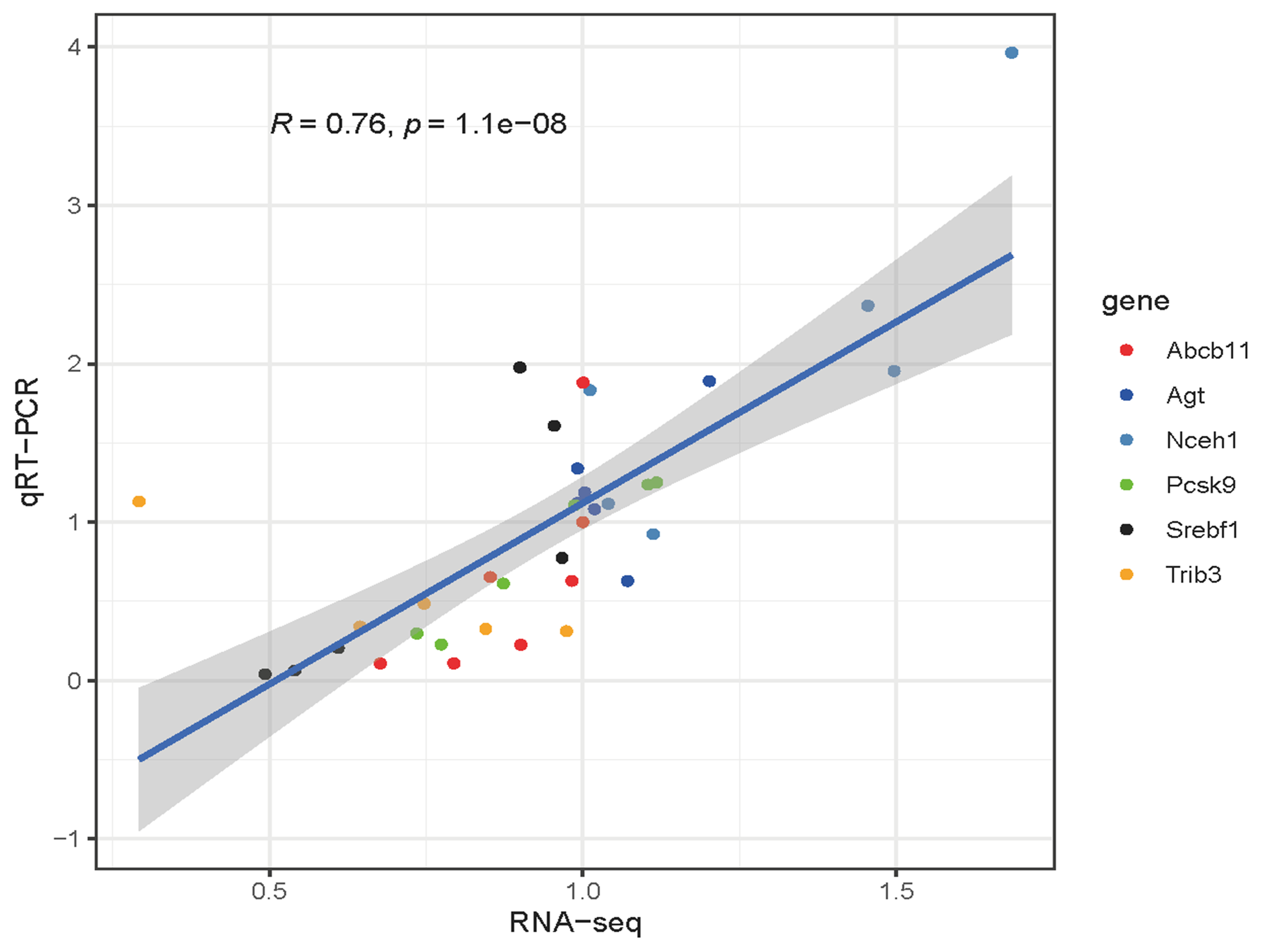

Supplement: Supplementary file 9 — Additional file 9: Figure S6. The pearson correlation analysis between qRT-PCR and RNA-seq. [file 12864_2023_9647_MOESM9_ESM.png]

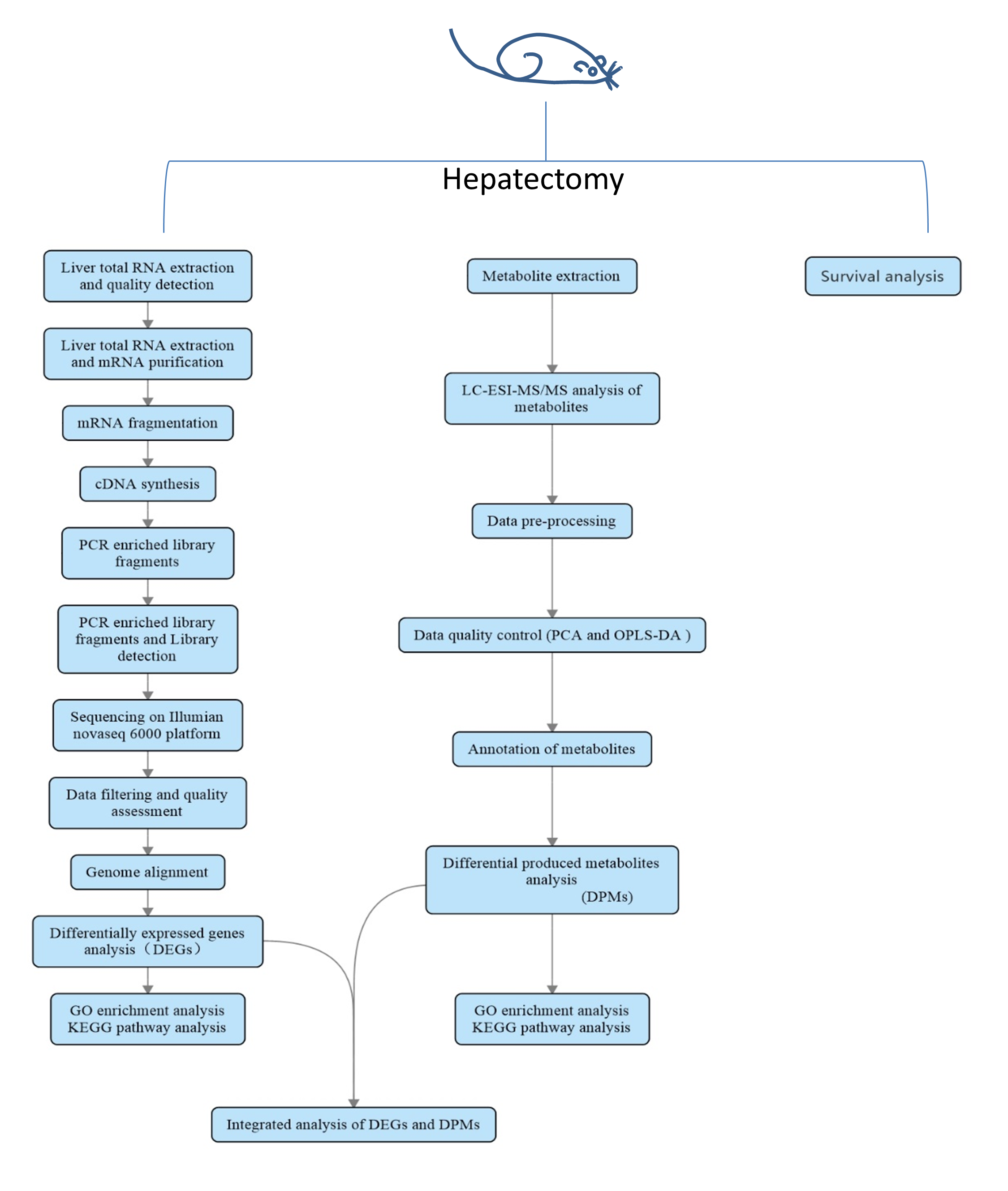

Supplement: Supplementary file 10 — Additional file 10: Figure S7. The experimental workflow chart of this study. [file 12864_2023_9647_MOESM10_ESM.png]
